# Risk seeking or averse, how do analyst coverage and firm visits motivate managers?

**DOI:** 10.1371/journal.pone.0328017

**Published:** 2025-07-11

**Authors:** Hangbo Liu, Xuemeng Guo, Dachen Sheng

**Affiliations:** 1 School of Economics and Management, Beijing Jiaotong University, Beijing, China; 2 International College of Liberal Arts, Yamanashi Gakuin University, Kofu, Yamanashi, Japan; 3 Department of Business & Economics, International Christian University, Mitaka, Tokyo, Japan; Bucharest University of Economic Studies: Academia de Studii Economice din Bucuresti, ROMANIA

## Abstract

In this research, we use Chinese stock exchange listed firm data to explore the relationship between institutional analysts’ firm visits and firms’ risk-taking as measured by earnings quality. The results show that more frequent visits in the previous year increase managers’ risk-taking decisions in later years and that firms experience lower earnings quality. Interestingly, the concentration of shareholding and management power, traditionally believed to be negative corporate governance instruments, alleviate such risk-taking if a firm is a state-owned enterprise (SOE). For non-SOEs, the more concentrated the shareholding and management power the further the risks increase. These results are attributed to the political connection of the manager, which would make managers more risk averse when under market focus and minimize risks that would damage their reputation and political career.

## Introduction

Financial analysts work for financial institutions to analyze the operating and financial conditions of firms [1]. Financial analysts give attention to a firm’s revenue growth, market share, operating and financial costs, and most importantly, earnings’ quality [2]. The firms disclose their financial reports following the accounting accrual methods. The accrual methods could better reflect the timely basis since the transaction happens, even before the flow of money occurs [3]. When performing financial analysis, analysts focus on how much revenue the firm can collect.

The collection of revenue or earning quality are used as the performance indicators that estimate firms’ future revenues by projecting current earnings to future dates and assuming the condition of the business environment [4]. Analysts may also assume good and bad periods in the future to perform scenario analysis. The results are then combined with the market premium condition to estimate the value of the firm in the financial market [5]. Some commonly used indicators include PE ratio and Tobin’s Q, which reflects the total value of the firm.

Managers’ attitude could be affected by financial analyst visits and coverage. In emerging markets, market and stock herding occur when the majority capital of investments and decisions are made by individual investors [6]. Rumors or news about a firm in the Chinese market are usually positive, since borrowing and short selling are extremely difficult [7,8], and analysts want to maintain good relationships with the firm, the targeting and the published analysis are usually positive [9]. Managers have high pay performance sensitivity since their reward is connected to firm share performance. When they realize that there is support for the share price since positive news would attract extra demand, managers have the incentive to take greater risk and seek higher performance. They could offer clients longer cash settlements, compete with other competitors for a larger market share by providing higher discounts and dealing with clients with lower credits, which could all negatively influence earning quality.

In this research, we choose the Chinese market as a candidate to explore the heterogeneities among managers of firms with different characteristics. The Chinese financial market is large and well developed compared with other emerging markets. Many exchanges have listed state-owned enterprises (SOEs) that bear both economic and social obligations. SOEs have significant shares owned by the government or government-related entities, and the managers of SOEs are assigned by the government or are politically well connected [10]. When making decisions, SOEs not only seek profit but also consider social benefits. Managers have their own career interests and need to execute government guidance well [11]. Such special personal interests could make economic-seeking decisions less efficient during normal decision-making, but they could alleviate aggressive economic-seeking agency costs and improve management and corporate governance by preventing managers from pursuing high-risk projects. Further, since the SOEs’ managers have close political connections or are directly assigned by the government shareholding entities, they may have strong risk-taking incentives compared to other well-diversified exchange-listed or family-oriented and controlled firms. The unique business and economic environment would allow us to test the different behaviors and better evaluate the impacts of the financial analysts’ visits upon firms and managers with different backgrounds.

This research provides a new direction for investigating the relationships between analysts’ coverage and managers’ behavior and decision-making in emerging markets. Past research findings of developed markets show that when there is a significant downturn in a firm’s earnings quality, the firm immediately engages more analysts to discover and explore the earnings of the firm [12]. Our finding differs from the use of concurrent information; we use past analysts’ firm visits and coverage changes to show that in emerging markets, managers could be motivated to actively seek greater risk, which could lower earnings quality, and such behavior is led by their confidence in future stabilized firm share prices.

## Literature review and hypotheses

Management and corporate governance are art and philosophy. The key concept of management is to put the right person in the right place, doing the appropriate task and making correct decisions. The firm’s board supervises the firm’s overall operations, and the CEO, as the general manager, makes specific decisions about operation and firm development strategies. From the operating aspect, higher board members and CEO education could increase intellectual capital efficiency and contribute to the firm growth [13,14]. Gender diversification could provide better supervision, which increases the liquidity and support from the banks and allows firms to rely more on debt financing [15]. The higher level of board independence and involvement of the institutional investors could reduce the business failure rate in emerging markets [16], and efficient corporate governance would reduce the information asymmetry and the potential conflicts between the shareholders and the management team [17].

### Theoretical frameworks

#### Principal agency problem.

The modern management and corporate governance suggest the separation between ownership and control [18]. The principal agency problem refers to the potential conflicts, like moral hazard, between managers and shareholders [19]. The theory states that managers should maximize the shareholders’ interest by behaving as the shareholders’ agent, but managers always tend to maximize their interests rather than consider the shareholders’ interests [20]. Such conflict leads the managers to choose inappropriate projects to invest in. Managers may seek higher returns and choose the larger risky project since managers receive a larger reward from the firm if the return is large, but the lower risk and moderate return project may best meet the shareholder’s target. Such a project increases the firm’s overall risk level.

#### Decision making.

From the management theory by Chester Barnard, managers often make cognitive and experienced decisions when they decide on new investments and expansions, which are complicated unstructured problems in management [21,22]. When managers receive external influence that they believe is positive for their firms, for example, the external report about the firm’s production, managers treat such report as a reaffirmation of their decision [23]. The managers could experience confirmation bias. Such a reaffirming feeling could make the managers overconfident and implement more aggressive but risky strategies [24]. Recent studies also note that the background of different managers in different environments may interpret a similar behaviour signal in completely different ways. Such heterogeneous understanding and behaviours are self-interest-related, so the reaction to the signals needs to be interpreted with the interest alignment and conflicts between managers and shareholders.

### Monitoring and corporate governance

Monitoring refers to supervising the managers’ behaviours and decisions in management and corporate governance [25]. The monitoring could significantly lower the agency costs. Monitoring could be internal and external. External monitoring often refers to auditing and social supervision, which ensures the financial reports meet the required quality criteria. Different management committees and shareholders do internal monitoring. For example, institutional shareholders often possess professional management experience, which is believed to be a positive sign of monitoring. They could intervene and supervise the firm performance to ensure the accounting quality and check the financial status to ensure the firm meets all loan covenant requirements from the creditors, which lowers the likelihood of financial manipulation [26]. Further, the use of debt by professional analysts from banks and other financial institutions to check the firm’s financial status would also lower any information asymmetry.

Management power should not be neglected when discussing monitoring, managers’ decisions, and the principal agency problem. This research shows managers experience confirmation bias when taking larger risks, but managers from state-owned enterprises (SOEs) would have different interests. Interestingly, the traditional negative management and corporate governance factor, the duality, which indicates excessive concentrated management power, would positively alleviate risk-taking from confirmation bias on SOEs managers.

### Analysts’ coverage in developed and emerging markets

Analyst coverage reflects the market focus, and more attention is given to regulating inappropriate management decisions, thereby improving corporate governance and lowering agency costs [27]. Financial analysts play a key role when firms issue additional equity [28]. There are different types of analysts working for different institutional investors and brokerage firms in the financial market. The analysts working for brokerage firms, known as sell-side analysts, simply share their opinions and ideas with the users who subscribe to their reports as the investors using the brokerage firm’s services [29], analyze the information from the firm and deliver it to investors [30,31]. The investors who subscribe to and make investment decisions based on those recommendations would increase the demand for the stocks they recommend, and more buy bids would increase the share price [32,33]. Managers who rely on share price and use it as part of their performance calculation indicator, to decide their compensation and reward as part of their pay performance contract, would be happy to see that their firm has been recommended to the public since it supports the share price, which increases the managers’ rewards.

In the developed market, managers would have stronger incentives to maximize shareholders’ interest since managers often hold the warrants and options of the firm’s shares as part of their wage rewards [34]. Even if imperfect, the instrument-like option could well control the managers’ incentive in the longer term, particularly if the share market volatility is moderate [35]. Managers would be less likely to deviate from the appropriate risk-reward investment and take a larger risk since the inappropriate risk may hurt the share price. It would affect the manager’s wealth from the options they hold. Further, the great coverage from the different financial institutions and their analysts would also monitor the manager’s behaviors [36].

In the emerging market, the managers may treat such a sell-side analyst’s recommendation as the qualified indicator as the confirmation signal which confirms their management ability [37]. Managers may cooperate with such market news to take higher risk projects to reflect the potential higher reward and to confirm with the sell-side analysts’ recommendations. Additionally, in most emerging markets, the share owned by institutional investors is usually a positive sign of high corporate governance quality since institutional investors have greater monitoring effects and have much more sophisticated knowledge of managing firms than individual investors do. When analysts from the buy side, for example, from mutual funds, visit the firm and investigate the details of the firm’s operation, the likelihood of mutual funds investing in the firm and becoming institutional shareholders becomes very high. Buy-side analysts may obtain information from published sell-side reports [38], but the focus of the research and understanding could be from different aspects [39]. Fund ownership is a good signal to the market and attracts more individual investors since, in emerging markets, many individual investors herd at the stock level to follow the fund or other more sophisticated investors because they believe that they have better knowledge [40], but those individual investors usually obtain negative abnormal return compared with the institutional investors, who lead herding to enjoy positive abnormal returns [41]. The followers of the individual investors increase the buy bids and increase the share price so that the managers can take a greater risk to further compete with other firms for larger market shares and provide more generous invoice payment conditions to the clients since they are less concerned about the firm’s share performance under such extra demand from the market [42]. With the logic above, we make the first hypothesis about manager behavior when more analysts from both the buy and sell sides covering and visiting the firm are observed.

H1. More coverage and visits from analysts increase managers’ risk-taking, which is reflected in later operating decisions.

### SOE management heterogeneity

This duality indicates that such a person possesses greater than usual management power when making operational decisions [43,44]. Of course, in economic theories, the risk-taking level would be dependent on the managers’ risk-averse level, even under duality [45], but since most managers believe that the firm’s shares would be invested with extra demand, and such extra demand would increase the share price or at least stabilize the share price if the firm is involved in negative news. This consideration may better fit shareholder diversified firms or more family-oriented firms in the Chinese market but not state-owned enterprises (SOEs).

SOEs could make different decisions with different operating targets and incentives [46]. When SOEs have duality, such a person would be a key person in making better politically connected decisions. Past research has shown that in non-Chinese markets, duality has negative effects [47]. Since most SOEs in the Chinese market are required to follow political guidance and intervention and have greater social responsibility rather than being merely economical, the key person’s decision would largely affect his future political career [48,49]. When there is more coverage and analysts following the firm, the firm is under greater focus, and any small mistake could be enlarged to be a negative political issue. Managers with greater management power would make extremely careful decisions and avoid any mistakes. Since SOEs are not purely seeking economic profit maximization, the manager would have no duty or interest in considering profitability performance first but would avoid any misjudgment [50]. If any decisions deteriorate earning quality, indicating greater future risk, the manager would then avoid such decisions and, on the other hand, try to improve earning quality and corporate governance quality when the firm is under market fucus [51]. With such logic, we propose our second hypothesis.

H2. More market coverage and analysts’ visits weaken the duality (large management power) of risk-taking in SOEs.

### Dominant shareholders and their structural influence on risk management

Share ownership and capital structure could significantly affect managers’ firm operating decisions [52,53]. The more concentrated shareholders’ structure may smooth the decision-making process, but from the corporate governance perspective, the monitor and other supervisions become weak when there are few independent board members and only a few larger shareholders control most of the shares [54,55]. The more diversified shareholder structure could allow for more shareholders with different expertise and therefore provide better monitoring of the firm’s operation, and the firm information disclosure is clear [56]. When managers of more concentrated firms feel that there is an opportunity, and if the firm takes some controllable risk to produce new products or take market shares, the firm may react quickly to change its strategy to reflect such considerations [57]. In SOEs, the largest shareholder is usually directly related to the local government. In addition to duality, some SOEs’ choices reflect political attitudes. Similar to the duality problem, SOEs are very careful when they face expansion decisions [58] and SOEs try to avoid making mistakes, with profitability performance not being the only goal. Similar to H2, we propose the following hypothesis.

H3. Analyst visits reduce the dominant shareholder risk-taking effects in SOEs.

## Data

This research uses the financial information of Chinese exchange listed firms between 2013 and 2023. Any firms from the finance sector are excluded since they have different accounting treatments. All firms’ financial statement information is collected from the Choice Database. The firms listed after the year 2014 and any financially distressed firms are also excluded. Earnings quality is used as a measurement indicator of manager risk-taking and is measured by the absolute value of the residual of equations (1) and (2) below [59,60]. Earnings quality refers to the likelihood of collecting accruals like account receivables in the future. After firms provide their services or products and recognize the revenue, the customers are invoiced, and the firm would need to collect the payments within the invoice period. There is a likelihood that the customer refuses or loses the ability to make payments, and in that case, the collection needs to go through a legal case, and the possibility of collection becomes questionable. Even though the accruals are inevitable, firms would mostly prefer cash or short-term invoice periods because the longer the waiting time, the higher the uncertainty. The accrual should grow proportionally with the change in total sales, but when the firm is seeking expansion or market competition, they may need to deal with lower credit customers or provide longer invoice periods to attract more customers, increasing the accruals. Sometimes, the manager could have an incentive to smooth or manage the earnings by recognizing the revenue and cost too fast or too slow, which could increase the volatility of the accruals, lowering the earning quality.


$${Accruals}_{i,t}=\alpha_{0}\frac{1}{{Asset}_{i,t-1}}+\alpha_{1}\frac{{CFO}_{i,t-1}}{{Asset}_{i,t-1}}+\alpha_{2}\frac{{CFO}_{i,t}}{{Asset}_{i,t-1}}+\alpha_{3}\frac{{CFO}_{i,t+1}}{{Asset}_{i,t-1}}+\alpha_{4}\frac{{\Delta sales}_{i,t}}{{Asset}_{i,t-1}}+\alpha_{5}\frac{{PPE}_{i,t}}{{Asset}_{i,t-1}}+\varepsilon_{i,t}$$ (1)

$${Accruals}_{i,t}=\beta_{0}+\beta_{1}{[\Delta Sales}_{i,t}-\Delta {AR}_{i,t}]+\beta_{2}{PPE}_{i,t}+\beta_{3}{ROA}_{i,t}+\varepsilon_{i,t}$$ (2)

Since equation (1) involves prior year and upcoming year operating cash flow (CFO), the actual financial information from the financial statements that matches the firm accruals is between 2014 and 2022. The descriptive statistics are shown in Table 1, and the variable definitions and treatments are shown in Table 2.

**Table 1:** Descriptive statistics.

Statistic	N	Mean	St. Dev.	Min	Max
ABS	11,943	1,323.296	3,451.151	0.266	88,083.940
RES	11,943	1,436.185	3,037.949	0.200	83,922.410
Liab	11,943	42.911	27.335	0.906	1,879.036
Current	11,943	2.290	2.743	0.074	76.458
EBITDA	11,943	0.252	0.450	−9.071	10.683
BPS	11,943	4.514	4.295	−10.954	157.226
EPS	11,943	0.332	1.178	−16.460	49.930
SOE	11,943	0.341	0.474	0	1
FIRST	11,943	31.385	13.853	1.840	89.990
DUALITY	11,943	0.243	0.429	0	1
Instvisit	11,943	22.791	63.713	0	1,448
Broker	11,943	5.078	9.018	0	104
Bank	11,943	0.217	1.059	0	23
Fund	11,943	5.430	13.360	0	171

**Table 2:** Variable definition and treatments.

Variable	Symbol	Variable Treatment
Earning quality measured by equation (1)	ABS	The absolute value of the residual of equation (1)
Earning quality measured by equation (2)	RES	The absolute value of the residual of equation (2), which is an alternative measure of variable ABS.
Liability ratio	Liab	Total liability/ Total asset
Current ratio	Current	Current asset/ Current liability
Earnings before interest, taxes, depreciation, and amortization	EBITDA	The observed earnings before interest, taxes, depreciation, and amortization from financial statements
Book value per share	BPS	Equity/ Total shares outstanding
Earning per share	EPS	Net income / Total shares outstanding
State-owned enterprise	SOE	Dummy variable, if the firm is state-owned enterprise, it equals 1
Percentage of total share held by largest shareholder	First	Largest shareholder shares / total shares
Manager and CEO is the same person	Duality	Dummy variable, if the firm manager and the chair of the board is the same person, it equals 1
Institutional investor visits	Instvisit	Number of institutional investor visits
Broker firm visits	Broker	Number of brokerage firm investor visits
Bank visits	Bank	Number of bank visits
Fund visit	Fund	Number of mutual fund analysts visits

## Methodologies

### Baseline model, analyst coverage in developed and emerging markets

The first set of tests involves the relationship between the visits performed in the prior year and the risk managers take after the visits, which is the baseline of all tests. As we described in the literature review section, equations (3) to (6) test the different relationships between visits by different institutions and how visits affect the earning quality or risk-taking of managers.


$${ABS}_{i,t}=\beta_{0}+\beta_{1}{Instvisit}_{i,t-1}+{\beta_{2}Liab}_{i,t}+{\beta_{3}BPS}_{i,t}+{\beta_{4}Current}_{i,t}+{\beta_{5}EPS}_{i,t}+{\beta_{6}EBITDA}_{i,t}+\sum IND+\sum YEAR+\varepsilon_{i,t}$$ (3)

$${ABS}_{i,t}=\beta_{0}+\beta_{1}{Broker}_{i,t-1}+{\beta_{2}Liab}_{i,t}+{\beta_{3}BPS}_{i,t}+{\beta_{4}Current}_{i,t}+{\beta_{5}EPS}_{i,t}+{\beta_{6}EBITDA}_{i,t}+\sum IND+\sum YEAR+\varepsilon_{i,t}$$ (4)

$${ABS}_{i,t}=\beta_{0}+\beta_{1}{Bank}_{i,t-1}+{\beta_{2}Liab}_{i,t}+{\beta_{3}BPS}_{i,t}+{\beta_{4}Current}_{i,t}+{\beta_{5}EPS}_{i,t}+{\beta_{6}EBITDA}_{i,t}+\sum IND+\sum YEAR+\varepsilon_{i,t}$$ (5)

$${ABS}_{i,t}=\beta_{0}+\beta_{1}{Fund}_{i,t-1}+{\beta_{2}Liab}_{i,t}+{\beta_{3}BPS}_{i,t}+{\beta_{4}Current}_{i,t}+{\beta_{5}EPS}_{i,t}+{\beta_{6}EBITDA}_{i,t}+\sum IND+\sum YEAR+\varepsilon_{i,t}$$ (6)

Then, similar regressions using the alternative measure of earning quality are performed to check the robustness of the results. Furthermore, the generalized method of moments is used to alleviate the endogeneities and to recheck the reliability of the baseline models.

### SOE management heterogeneity

The second set of tests concerns how duality affects SOEs and non-SOEs after different visits are performed. The focus shifts to the interactive term in equations (7) to (10). The samples are divided into subsamples on the basis of whether the firm is an SOE or a nonSOE; then, regressions are performed to observe the interaction term coefficients.


$$\begin{array}{c} {ABS}_{i,t}=\beta_{0}+\beta_{1}{Instvisit}_{i,t-1}+\beta_{2}{Duality}_{i,t}+{\beta_{3}Liab}_{i,t}+{\beta_{4}BPS}_{i,t}+{\beta_{5}Current}_{i,t}+{\beta_{6}EPS}_{i,t}+\beta_{7}{EBITDA}_{i,t}\\ +\beta_{8}\left[{Instvisit}_{i,t-1}*{Duality}_{i,t}\right]+\sum IND+\sum YEAR+\varepsilon_{i,t} \end{array}$$ (7)

$$\begin{array}{c} {ABS}_{i,t}=\beta_{0}+\beta_{1}{Broker}_{i,t-1}+\beta_{2}{Duality}_{i,t}+{\beta_{3}Liab}_{i,t}+{\beta_{4}BPS}_{i,t}+{\beta_{5}Current}_{i,t}+{\beta_{6}EPS}_{i,t}+\beta_{7}{EBITDA}_{i,t}\\ +\beta_{8}[{Broker}_{i,t-1}*{Duality}_{i,t}]+\sum IND+\sum YEAR+\varepsilon_{i,t} \end{array}$$ (8)

$$\begin{array}{c} {ABS}_{i,t}=\beta_{0}+\beta_{1}{Bank}_{i,t-1}+\beta_{2}{Duality}_{i,t}+{\beta_{3}Liab}_{i,t}+{\beta_{4}BPS}_{i,t}+{\beta_{5}Current}_{i,t}+{\beta_{6}EPS}_{i,t}+\beta_{7}{EBITDA}_{i,t}\\ +\beta_{8}\left[{Bank}_{i,t-1}*{Duality}_{i,t}\right]+\sum IND+\sum YEAR+\varepsilon_{i,t} \end{array}$$ (9)

$$\begin{array}{c} {ABS}_{i,t}=\beta_{0}+\beta_{1}{Fund}_{i,t-1}+\beta_{2}{Duality}_{i,t}+{\beta_{3}Liab}_{i,t}+{\beta_{4}BPS}_{i,t}+{\beta_{5}Current}_{i,t}+{\beta_{6}EPS}_{i,t}+\beta_{7}{EBITDA}_{i,t}\\ +\beta_{8}\left[{Fund}_{i,t-1}*{Duality}_{i,t}\right]+\sum IND+\sum YEAR+\varepsilon_{i,t} \end{array}$$ (10)

### Dominant shareholders and their structural influence on risk management

The third set of tests focuses on the dominant shareholder effect rather than duality. Similar test methods are used. The sample is still divided into two subsamples: an SOE subsample and a non-SOE subsample. The variable “Duality” is replaced by the variable “First”, and the same regressions of equations (7) to (10) are retested to observe the interactive term coefficients.

## Results

### Baseline model: analyst coverage in developed and emerging markets

The baseline model results are shown in Table 3. All of the visit variables have significant positive coefficients. The finding indicates that a greater number of visits increases risk-taking and decreases the earning quality of a firm. Our results confirm that overconfident managers are making more aggressive decisions [61]. The results are also in line with the past point that the manager’s overconfidence is a rational reaction to the economic and institutional environment [62]. Analysts’ visits, particularly from buy-side analysts, increase the confidence of firm managers and motivate them to take slightly greater risk. Managers understand the effect of buy-side investments and the positive reputation and share price support effect they can enjoy. Therefore, even if we always treat institutional ownership as a good signal for corporate finance, particularly if we argue that it can be carefully monitored, but we ignore the effect that before the institutional investor invests in the firm, the investigation and understanding of the business could increase the degree of risk faced by the firm.

**Table 3:** Visits and earning quality.

	*Dependent variable*:			
	ABS			
	(1)	(2)	(3)	(4)
${Instvisit}_{t-1}$	6.621^***^			
	(0.488)			
${Broker}_{t-1}$		36.433^***^		
		(3.496)		
${Bank}_{t-1}$			678.028^***^	
			(28.604)	
${Fund}_{t-1}$				20.091^***^
				(2.355)
Liab	14.903^***^	15.113^***^	14.215^***^	14.887^***^
	(1.262)	(1.266)	(1.243)	(1.268)
BPS	166.439^***^	164.404^***^	161.389^***^	167.802^***^
	(12.171)	(12.223)	(11.986)	(12.230)
Current	−34.421^***^	−34.369^***^	−30.209^**^	−36.070^***^
	(12.938)	(12.982)	(12.741)	(12.998)
EPS	−238.087^***^	−222.902^***^	−235.205^***^	−232.702^***^
	(46.608)	(46.740)	(45.883)	(46.825)
EBITDA	109.170	102.943	117.807	122.178
	(83.508)	(83.866)	(82.162)	(83.930)
Constant	−269.408	−320.039	−183.327	−298.222
	(861.178)	(863.871)	(847.997)	(865.166)
IND CONTROL	Y	Y	Y	Y
YEAR CONTROL	Y	Y	Y	Y
Observations	11,943	11,943	11,943	11,943
R^2^	0.088	0.082	0.116	0.080
Adjusted R^2^	0.083	0.077	0.111	0.074
Residual Std. Error (df = 11874)	3,304.833	3,315.185	3,254.202	3,320.154
F Statistic (df = 68; 11874)	16.895^***^	15.701^***^	22.900^***^	15.131^***^

*Note:* ^*^p^**^p^***^p < 0.01

In Table 4, the measurement of earning quality has been changed. In Table 4, all of the visit coefficients are positively significant, which indicates that a greater number of visits increases the degree of risk that managers take and lowers earning quality. These results confirm our baseline model results and show the robustness of the original results and relationships. Table 5 involves the use of GMM methods to recheck the reliability of the results to alleviate any potential endogeneity problems, even when lagged visits are used as the interest variable. After adding the lagged dependent variable in the GMM, almost all visits are significant except for the Bank variable. The results show the reliability of the original baseline model and support our Hypothesis H1.

**Table 4:** Visits and earning quality remeasurement.

	*Dependent variable:*			
	RES			
	(1)	(2)	(3)	(4)
${Instvisit}_{t-1}$	4.113^***^			
	(0.433)			
${Broker}_{t-1}$		20.015^***^		
		(3.097)		
${Bank}_{t-1}$			461.177^***^	
			(25.508)	
${Fund}_{t-1}$				10.170^***^
				(2.085)
Liab	11.770^***^	11.874^***^	11.312^***^	11.743^***^
	(1.119)	(1.122)	(1.108)	(1.122)
BPS	139.857^***^	139.133^***^	136.099^***^	141.177^***^
	(10.792)	(10.827)	(10.689)	(10.825)
Current	−3.642	−3.924	−0.521	−4.974
	(11.473)	(11.499)	(11.362)	(11.505)
EPS	−173.165^***^	−163.702^***^	−172.126^***^	−168.644^***^
	(41.330)	(41.401)	(40.917)	(41.446)
EBITDA	31.715	32.013	34.532	44.255
	(74.051)	(74.287)	(73.270)	(74.288)
Constant	158.126	127.349	219.111	138.799
	(763.653)	(765.198)	(756.219)	(765.777)
IND CONTROL	Y	Y	Y	Y
YEAR CONTROL	Y	Y	Y	Y
Observations	11,943	11,943	11,943	11,943
R^2^	0.075	0.071	0.093	0.070
Adjusted R^2^	0.069	0.066	0.087	0.064
Residual Std. Error (df = 11874)	2,930.573	2,936.519	2,902.006	2,938.736
F Statistic (df = 68; 11874)	14.105^***^	13.341^***^	17.839^***^	13.058^***^

*Note:* ^*^p^**^p^***^p < 0.01

**Table 5:** GMM method.

	*Dependent variable:*			
	ABS			
	(1)	(2)	(3)	(4)
lag(ABS, 1)	0.329^***^	0.330^***^	0.326^***^	0.331^***^
	(0.050)	(0.050)	(0.050)	(0.050)
${Instvisit}_{t-1}$	0.948^***^			
	(0.221)			
${Broker}_{t-1}$		3.278^***^		
		(1.242)		
${Bank}_{t-1}$			22.490	
			(15.129)	
${Fund}_{t-1}$				3.090^***^
				(0.845)
Liab	−0.377	−0.374	−0.367	−0.376
	(0.613)	(0.615)	(0.618)	(0.615)
Current	−5.003	−4.777	−4.557	−4.932
	(3.683)	(3.706)	(3.692)	(3.679)
BPS	23.538^**^	24.527^**^	24.629^**^	23.415^**^
	(11.669)	(11.740)	(11.761)	(11.698)
EBITDA	−55.496^**^	−55.408^**^	−55.690^**^	−54.892^**^
	(26.202)	(26.357)	(26.345)	(26.207)
EPS	54.566^**^	54.570^*^	55.195^**^	54.235^**^
	(27.566)	(28.092)	(28.110)	(27.569)
IND Control	Y	Y	Y	Y
Year Control	Y	Y	Y	Y
Observations	1,327	1,327	1,327	1,327

*Note:* ^*^p^**^p^***^p < 0.01

### SOE management heterogeneity

In Table 6 and Table 7, the results show the heterogeneities when firm characteristics are different. In Table 6, the subsamples are all SOEs, and visits still have significant positive coefficients. However, when a firm has a strong manager, strong management power reduces risk-taking, and all of the visit and duality interaction terms have negative significant coefficients. Our findings are in line with the literatures about the Chinese SOEs social obligations [63], and the SOEs’ CSR (corporate social responsibility) attitude and practice to closely follow the policy guidance [64]. These results show that the manager with the largest power, who is usually the assigned officer to work in the firm or the one with strong political connections, is more risk averse. Rather than seeking higher potential returns, the manager chooses to lower the risk when the firm is under the focus of the public or covered by a large number of analysts.

**Table 6:** SOE firms’ duality effect on visits.

	*Dependent variable:*			
	ABS			
	(1)	(2)	(3)	(4)
${Instvisit}_{t-1}$	11.484^***^			
	(1.152)			
${Broker}_{t-1}$		72.605^***^		
		(9.441)		
${Bank}_{t-1}$			835.681^***^	
			(68.186)	
${Fund}_{t-1}$				48.066^***^
				(6.778)
Duality	54.992	115.791	3.365	35.638
	(275.516)	(295.022)	(268.649)	(285.097)
Liab	40.769^***^	40.850^***^	40.450^***^	40.618^***^
	(4.775)	(4.800)	(4.745)	(4.804)
BPS	130.647^***^	128.395^***^	133.144^***^	128.876^***^
	(26.697)	(26.862)	(26.513)	(26.900)
Current	66.407	71.406	75.827^*^	65.811
	(44.484)	(44.699)	(44.202)	(44.757)
EPS	−268.671^***^	−258.133^***^	−277.643^***^	−262.974^***^
	(98.276)	(98.791)	(97.620)	(98.914)
EBITDA	299.676	298.320	349.345	301.934
	(247.775)	(249.205)	(246.022)	(249.528)
${Instvisit}_{t-1}$^*^Duality	−10.652^***^			
	(3.535)			
${Broker}_{t-1}$^*^Duality		−67.860^**^		
		(28.215)		
${Bank}_{t-1}$^*^Duality			−676.958^***^	
			(224.493)	
${Fund}_{t-1}$^*^Duality				−42.705^**^
				(19.708)
Constant	−2,087.999	−2,176.776	−2,067.646	−2,099.979
	(1,570.727)	(1,578.460)	(1,561.054)	(1,580.185)
IND CONTROL	Y	Y	Y	Y
YEAR CONROL	Y	Y	Y	Y
Observations	4,078	4,078	4,078	4,078
R^2^	0.115	0.107	0.126	0.105
Adjusted R^2^	0.103	0.094	0.114	0.092
Residual Std. Error (df = 4020)	4,581.387	4,603.979	4,553.169	4,608.959
F Statistic (df = 57; 4020)	9.199^***^	8.419^***^	10.191^***^	8.248^***^

*Note:* ^*^p^**^p^***^p < 0.01

**Table 7:** NonSOE firms duality effect on visits.

	*Dependent variable:*			
	ABS			
	(1)	(2)	(3)	(4)
${Instvisit}_{t-1}$	3.685^***^			
	(0.611)			
${Broker}_{t-1}$		19.830^***^		
		(3.673)		
${Bank}_{t-1}$			605.047^***^	
			(32.984)	
${Fund}_{t-1}$				7.665^***^
				(2.499)
Duality	−47.748	−110.807^*^	−32.489	−74.521
	(60.238)	(66.730)	(55.113)	(61.224)
Liab	9.566^***^	9.687^***^	8.929^***^	9.591^***^
	(0.946)	(0.946)	(0.918)	(0.948)
BPS	195.975^***^	193.171^***^	186.113^***^	198.444^***^
	(11.335)	(11.372)	(10.992)	(11.370)
Current	−24.244^**^	−23.943^**^	−20.007^**^	−25.525^**^
	(10.022)	(10.032)	(9.721)	(10.052)
EPS	153.844^***^	168.022^***^	132.746^***^	164.606^***^
	(46.770)	(46.715)	(45.300)	(46.918)
EBITDA	−162.962^**^	−172.641^**^	−154.518^**^	−159.754^**^
	(69.235)	(69.307)	(67.153)	(69.446)
${Instvisit}_{t-1}$^*^Duality	0.786			
	(0.838)			
${Broker}_{t-1}$^*^Duality		13.984^**^		
		(5.763)		
${Bank}_{t-1}$^*^Duality			−23.687	
			(48.594)	
${Fund}_{t-1}$^*^Duality				9.001^**^
				(3.702)
Constant	392.918	442.823	478.145	395.753
	(927.293)	(928.251)	(899.480)	(930.153)
IND CONTROL	Y	Y	Y	Y
YEAR CONTROL	Y	Y	Y	Y
Observations	7,865	7,865	7,865	7,865
R^2^	0.120	0.119	0.172	0.115
Adjusted R^2^	0.113	0.112	0.165	0.107
Residual Std. Error (df = 7797)	2,255.821	2,257.089	2,188.456	2,262.645
F Statistic (df = 67; 7797)	15.914^***^	15.766^***^	24.184^***^	15.118^***^

*Note:* ^*^p^**^p^***^p < 0.01

In Table 7, the results show that when firms are nonSOEs, greater concentrated management power does not reduce but rather increases risk-taking. The visit terms still all have positive significant coefficients, and the two important visits, broker and fund, which typically represent the buy-side and the sell-side analysts, increase the managers’ risk-taking. These results supported our Hypothesis H2.

### Dominant shareholders and their structural influence on risk management

Table 8 and Table 9 show how the largest shareholder or the shareholder structure affects visits’ risk-taking motivation. n Table 8, all the visits have significant positive coefficients, which indicates that more visits increase the risk taken. However, the interaction terms between different visits and the First, which is the largest shareholder holding percentage, all have significant negative coefficients. These results show that when firms are SOEs, a more concentrated share structure or providing the largest shareholder with larger shares would reduce risk-taking and increase earnings quality. Our finding aligns with the literature’s point that the SOEs in the Chinese market are more conservative, both on management and strategies [65].

**Table 8:** SOE firms largest shareholder influence on earning quality.

	*Dependent variable:*			
	ABS			
	(1)	(2)	(3)	(4)
${Instvisit}_{t-1}$	28.030^***^			
	(2.948)			
${Broker}_{t-1}$		230.052^***^		
		(21.477)		
${Bank}_{t-1}$			2,409.289^***^	
			(169.189)	
${Fund}_{t-1}$				129.346^***^
				(15.571)
FIRST	69.633^***^	78.772^***^	68.839^***^	71.103^***^
	(5.750)	(5.992)	(5.551)	(5.876)
Liab	40.021^***^	40.193^***^	38.558^***^	40.042^***^
	(4.693)	(4.698)	(4.629)	(4.722)
BPS	93.499^***^	88.656^***^	96.439^***^	90.902^***^
	(26.409)	(26.469)	(26.018)	(26.609)
Current	60.984	63.891	65.908	59.775
	(43.697)	(43.743)	(43.089)	(43.971)
EPS	−206.217^**^	−187.985^*^	−217.617^**^	−198.574^**^
	(96.675)	(96.822)	(95.331)	(97.288)
EBITDA	110.028	101.824	156.519	120.870
	(243.902)	(244.381)	(240.275)	(245.645)
${Instvisit}_{t-1}$^*^First	−0.510^***^			
	(0.081)			
${Broker}_{t-1}$^*^First		−4.769^***^		
		(0.583)		
${Bank}_{t-1}$^*^First			−44.441^***^	
			(4.254)	
${Fund}_{t-1}$^*^First				−2.582^***^
				(0.445)
Constant	−4,968.074^***^	−5,433.213^***^	−4,842.125^***^	−5,055.054^***^
	(1,561.412)	(1,564.685)	(1,539.165)	(1,571.602)
IND CONTROL	Y	Y	Y	Y
YEAR CONTROL	Y	Y	Y	Y
Observations	4,078	4,078	4,078	4,078
R^2^	0.146	0.144	0.170	0.136
Adjusted R^2^	0.134	0.132	0.158	0.124
Residual Std. Error (df = 4020)	4,500.333	4,505.418	4,438.095	4,527.962
F Statistic (df = 57; 4020)	12.097^***^	11.911^***^	14.431^***^	11.092^***^

*Note:* ^*^p^**^p^***^p < 0.01

**Table 9:** NonSOE firms largest shareholder influence on earning quality.

	*Dependent variable:*			
	ABS			
	(1)	(2)	(3)	(4)
${Instvisit}_{t-1}$	1.559			
	(1.015)			
${Broker}_{t-1}$		16.892^**^		
		(6.681)		
${Bank}_{t-1}$			551.007^***^	
			(50.310)	
${Fund}_{t-1}$				0.301
				(4.363)
FIRST	−5.091^**^	−4.489^*^	−2.361	−5.319^**^
	(2.202)	(2.398)	(1.993)	(2.217)
Liab	9.535^***^	9.679^***^	8.925^***^	9.572^***^
	(0.945)	(0.946)	(0.918)	(0.948)
BPS	196.624^***^	193.804^***^	186.431^***^	199.155^***^
	(11.330)	(11.379)	(10.994)	(11.371)
Current	−24.502^**^	−23.891^**^	−20.428^**^	−25.569^**^
	(10.017)	(10.033)	(9.722)	(10.050)
EPS	152.728^***^	169.233^***^	134.144^***^	163.600^***^
	(46.820)	(46.787)	(45.358)	(46.982)
EBITDA	−157.134^**^	−168.856^**^	−150.423^**^	−153.386^**^
	(69.264)	(69.381)	(67.214)	(69.501)
${Instvisit}_{t-1}$^*^First	0.094^***^			
	(0.034)			
${Broker}_{t-1}$^*^First		0.290		
		(0.209)		
${Bank}_{t-1}$^*^First			1.743	
			(1.805)	
${Fund}_{t-1}$^*^First				0.406^***^
				(0.141)
Constant	528.055	494.488	533.254	511.482
	(928.322)	(929.950)	(900.669)	(931.351)
IND CONTROL	Y	Y	Y	Y
YEAR CONTROL	Y	Y	Y	Y
Observations	7,865	7,865	7,865	7,865
R^2^	0.121	0.119	0.172	0.115
Adjusted R^2^	0.114	0.111	0.165	0.108
Residual Std. Error (df = 7797)	2,254.647	2,257.426	2,188.288	2,262.059
F Statistic (df = 67; 7797)	16.052^***^	15.726^***^	24.205^***^	15.186^***^

*Note:* ^*^p^**^p^***^p < 0.01

In Table 9, when firms are nonSOEs, visits still have a positive coefficient, except for funding; all other variables are still significant, which shows that visits generally increase risk-taking. The interactive terms, however, have a positive coefficient, with the fund and overall institutional investor visits coefficients being significant. These results show that a more concentrated share structure with a larger largest shareholder usually increases risk-taking when analysts have many visits. These results support our Hypothesis H3.

### Discussion

The empirical evidence of the baseline models shows a stable negative relationship between past recent institutional analyst visits and earning qualities; more frequent visits decrease earnings quality; hence, managers take greater risks. These findings are in line with the downturn in earning quality attracting immediate analyst coverage in developed markets. Since even investors are allowed to short sell equity in the Chinese market, it is extremely difficult to find brokerage firms that are willing to lend shares [66]. More importantly, analysts usually do not explore negative stories since they want to maintain good relationships with firms so that firms can host their future visits.

Our study differentiates from past studies by Gao et al. (2022) [67], which focuses on the sell side analysts’ visit, providing the monitoring effect. Their study focuses on the current effect, but our study relies on last year’s visit performed by the analysts to forecast the managers’ risk-taking in the next year. Further, the sample period of our study covers much later years, and the financial market has significantly developed, which changes the primarily sell-side analysts to the mixture of buy, sell and analysts from financial intermediations. However, the results of the SOEs firms with duality in our study align with their results, which shows the analysts’ coverage would be an efficient monitoring instrument for the SOEs when their manager cares about their future political careers.

Our work also differentiates from Gao et al. (2023) [68], a study about the earning forecast disclosure associated with the analyst visits from the information asymmetry aspect. They focus on how the analysts’ visit could reduce the information asymmetry in quarterly time. As mentioned earlier in the literature review section, the managers would be incentivized to provide positive information as the analysts visit, and since the analysts in earlier times only issued positive analyst reports in the Chinese market to keep good relationships with the firms. Also, the study by Cheng et al. (2016) [69] shows that information acquisition could be improved if the analyst performs the firm visit. The market investors, particularly the individual investors, would trust the reports of the site-visited analysts more, and the positive reports would push up the share price. The increase in wealth after the managers anticipate the change in the financial market in the later year could increase their risk-taking incentives.

SOEs closely follow political requirements and guidance in the Chinese market [70]. Both duality and more concentrated largest shareholder holding indicate greater official influence, since if the manager is assigned by the government, the manager must follow the government decisions. When they are under public scrutiny, they would avoid making any mistakes so that reducing the risk would be a smart move. Even if nonSOE CEOs with more political connections lower their career risk [71], when they make operation decisions, when profit maximization firms realize there is good news related to them issued by financial analysts, they take additional risks and seek higher returns to confirm to the market the validity of their good news, especially when decision power is concentrated. From such a point, the concentrated structure is not always negative, particularly for SOEs. The concentrated power, which indicates deeper political connections, makes managers more risk averse.

Our findings show that the external environment could easily influence managers in emerging markets, and managers treat them as the confirmation of their management success, leading to more aggressive strategies. Such behaviour could increase the shareholder’s agency costs. However, the SOEs managers are less affected by such influence, and this is because the larger management power of SOEs managers would also indicate larger responsibilities. The risk of project failure would put the career at risk, which leads SOEs managers to make more conservative decisions. Such constraints interestingly reverse the most often negative concentrated management power as a factor that alleviates confirmation bias among SOEs in the Chinese market.

From the policy perspective, the regulation may encourage financial analysts to perform multiple visits and follow-ups once they finish the reports, particularly if the firm is non-SOEs. From the literature, the current visit could regulate the managers’ behaviors and reduce the risk-taking, but we have shown the visit, and once the managers observe the market demand changes, it increases their risk-taking. The known future revisits would be a valid threat that could help regulate the manager’s risk-taking behaviors. Further, the regulation authorities may also encourage the analysts to keep high ethical standards to provide better service to the investors.

## Conclusion

In this research, we have shown that firm visits by institutional financial analysts increases managers’ risk-taking incentives, which is reflected by a lower level of future earnings quality. Furthermore, we argue that even though higher large shareholder share concentration and management power duality are traditionally negative in corporate governance, they reduce the risk-taking behaviors of SOEs when the firm receives greater financial analyst coverage. The reason is that a greater concentration of share or management power indicates that CEOs have deeper political connections. Any mistake increases the risk to CEOs’ political career, so they choose to reduce rather than increase risk-taking.

Our findings have high policy value. The policy-maker should consider such management power, market coverage, and excessive risk-taking relationships. Analysts’ coverage could be an efficient instrument for monitoring SOEs. However, in the developing market, greater analyst coverage may not be an appropriate nonSOE monitoring instrument, since when managers have strong economic or profit maximization interest, they could increase rather than decrease risk-taking, especially if managers have a reward contract with high pay performance sensitivity. As we have recommended in the discussion section, if the regulation requires analysts to follow up on their reports and make updates by performing firm visits multiple times, the strategy may reduce the willingness of the managers to take on higher risks. The analysts should self-regulate and maintain high ethical standards to reject issuing any misleading reports or under-the-table deals.

## Supporting information

S1 File Data and code. (ZIP)
