# Supplementary material for: Risk seeking or averse, how do analyst coverage and firm visits motivate managers?
Source: PLoS One. 2025 Jul 11;20(7):e0328017. doi: 10.1371/journal.pone.0328017 (PMC12250548; doi:10.1371/journal.pone.0328017)
Supplement: S1 File — (ZIP) [file pone.0328017.s001.zip › R code do file.docx]

reg1<-lm(abresidual~Linstvisit+liab+BPS+current+EPS+EBITDA+ind+year,data=data)

summary(reg1)

regF1<-lm(abresidual~Lbroker+liab+BPS+current+EPS+EBITDA+ind+year,data=data)

summary(regF1)

regF11<-lm(abresidual~Lbank+liab+BPS+current+EPS+EBITDA+ind+year,data=data)

summary(regF11)

regF111<-lm(abresidual~Lfund+liab+BPS+current+EPS+EBITDA+ind+year,data=data)

summary(regF111)

rega1<-lm(abresidual~instvisit*DINST+liab+BPS+current+EPS+EBITDA+ind+year,data=data)

summary(rega1)

rega11<-lm(RES~instvisit*DINST+liab+BPS+current+EPS+EBITDA+ind+year,data=data)

summary(rega11)

reg11<-lm(RES~Linstvisit+liab+BPS+current+EPS+EBITDA+ind+year,data=data)

summary(reg11)

regNF1<-lm(RES~Lbroker+liab+BPS+current+EPS+EBITDA+ind+year,data=data)

summary(regNF1)

regNF11<-lm(RES~Lbank+liab+BPS+current+EPS+EBITDA+ind+year,data=data)

summary(regNF11)

regNF111<-lm(RES~Lfund+liab+BPS+current+EPS+EBITDA+ind+year,data=data)

summary(regNF111)

library(plm)

z01 <- pgmm(abresidual~lag(abresidual,1)+Linstvisit+liab+current+BPS+EBITDA+EPS

| lag(abresidual, 2:99),

index=c("name","year"), data = data, effect = "twoways", model = "twosteps")

summary(z01, robust = FALSE)

z02 <- pgmm(abresidual~lag(abresidual,1)+Lbroker+liab+current+BPS+EBITDA+EPS

| lag(abresidual, 2:99),

index=c("name","year"), data = data, effect = "twoways", model = "twosteps" )

summary(z02, robust = FALSE)

z03 <- pgmm(abresidual~lag(abresidual,1)+Lbank+liab+current+BPS+EBITDA+EPS

| lag(abresidual, 2:99),

index=c("name","year"), data = data, effect = "twoways", model = "twosteps")

summary(z03, robust = FALSE)

z04 <- pgmm(abresidual~lag(abresidual,1)+Lfund+liab+current+BPS+EBITDA+EPS

| lag(abresidual, 2:99),

index=c("name","year"), data = data, effect = "twoways", model = "twosteps")

summary(z04, robust = FALSE)

SOE<-subset(data,SOE==1)

regSOE1<-lm(abresidual~Linstvisit*DUALITY+liab+BPS+current+EPS+EBITDA+ind+year,data=SOE)

summary(regSOE1)

regSOE2<-lm(abresidual~Lbroker*DUALITY+liab+BPS+current+EPS+EBITDA+ind+year,data=SOE)

summary(regSOE2)

regSOE3<-lm(abresidual~Lbank*DUALITY+liab+BPS+current+EPS+EBITDA+ind+year,data=SOE)

summary(regSOE3)

regSOE4<-lm(abresidual~Lfund*DUALITY+liab+BPS+current+EPS+EBITDA+ind+year,data=SOE)

summary(regSOE4)

NSOE<-subset(data,SOE==0)

regNSOE1<-lm(abresidual~Linstvisit*DUALITY+liab+BPS+current+EPS+EBITDA+ind+year,data=NSOE)

summary(regNSOE1)

regNSOE2<-lm(abresidual~Lbroker*DUALITY+liab+BPS+current+EPS+EBITDA+ind+year,data=NSOE)

summary(regNSOE2)

regNSOE3<-lm(abresidual~Lbank*DUALITY+liab+BPS+current+EPS+EBITDA+ind+year,data=NSOE)

summary(regNSOE3)

regNSOE4<-lm(abresidual~Lfund*DUALITY+liab+BPS+current+EPS+EBITDA+ind+year,data=NSOE)

summary(regNSOE4)

regFSOE1<-lm(abresidual~Linstvisit*FIRST+liab+BPS+current+EPS+EBITDA+ind+year,data=SOE)

summary(regFSOE1)

regFSOE2<-lm(abresidual~Lbroker*FIRST+liab+BPS+current+EPS+EBITDA+ind+year,data=SOE)

summary(regFSOE2)

regFSOE3<-lm(abresidual~Lbank*FIRST+liab+BPS+current+EPS+EBITDA+ind+year,data=SOE)

summary(regFSOE3)

regFSOE4<-lm(abresidual~Lfund*FIRST+liab+BPS+current+EPS+EBITDA+ind+year,data=SOE)

summary(regFSOE4)

regFNSOE1<-lm(abresidual~Linstvisit*FIRST+liab+BPS+current+EPS+EBITDA+ind+year,data=NSOE)

summary(regFNSOE1)

regFNSOE2<-lm(abresidual~Lbroker*FIRST+liab+BPS+current+EPS+EBITDA+ind+year,data=NSOE)

summary(regFNSOE2)

regFNSOE3<-lm(abresidual~Lbank*FIRST+liab+BPS+current+EPS+EBITDA+ind+year,data=NSOE)

summary(regFNSOE3)

regFNSOE4<-lm(abresidual~Lfund*FIRST+liab+BPS+current+EPS+EBITDA+ind+year,data=NSOE)

summary(regFNSOE4)
